# Research priorities for adult hospital medicine: A survey of US hospital medicine leaders

**DOI:** 10.1002/jhm.70053

**Published:** 2025-04-20

**Authors:** Valerie G. Press, James D. Harrison, Sagar B. Dugani, Blair P. Golden, Nicolas Caravelli, Andrea Jackson-Sagredo, Valerie M. Vaughn, Andrew McWilliams, Juliessa M. Pavon, Ashley M. Jenkins, Andrew Sumarsono, Maylyn Martinez, Micah Prochaska, Stephanie K. Mueller

**Affiliations:** 1 Section of General Internal Medicine, Department of Medicine, University of Chicago, Chicago, Illinois, USA; 2 Division of Hospital Medicine, University of California San Francisco, San Francisco, California, USA; 3 Division of Hospital Internal Medicine, Mayo Clinic, Rochester, Minnesota, USA; 4 Robert D. and Patricia E. Kern Center for the Science of Health Care Delivery, Mayo Clinic, Rochester, Minnesota, USA; 5 Division of Hospital Medicine, University of Wisconsin School of Medicine and Public Health, Madison, Wisconsin, USA; 6 Department of Internal Medicine, Division of General Internal Medicine, University of Utah School of Medicine, Salt Lake City, Utah, USA; 7 Department of Internal Medicine, Division of Hospital Medicine, Wake Forest University School of Medicine, Winston-Salem, North Carolina, USA; 8 Department of Medicine, Division of Geriatrics, Duke University School of Medicine, Durham, North Carolina, USA; 9 Department of Medicine, Pediatrics, University of Rochester School of Medicine and Dentistry, Rochester, New York, USA; 10 Division of Hospital Medicine, University of Texas Southwestern Medical Center, Dallas, Texas, USA; 11 Section of Hospital Medicine, Department of Medicine, University of Chicago, Chicago, Illinois, USA; 12 Division of General Internal Medicine, Brigham and Women’s Hospital, Boston, Massachusetts, USA

## Abstract

Hospital medicine (HM), a well-established clinical specialty, requires clarity of research priorities to identify target areas for investment in HM research infrastructure and activities. The Society of Hospital Medicine’s Research Committee developed a 20-item survey and used purposeful sampling of US hospitalist leaders to prioritize primary research topic domains and subdomains. Respondents were asked to rank their HM research priorities using a scale (1 [highest] to 8 [lowest]). Of 239 surveys distributed, 81 (34%) responded. Respondents were predominantly White (53%) and from academic institutions (57%). “Overall, 46% of respondents were male and 33% were female.” Top-ranked research domains were innovation in care delivery (median 2, IQR 1,3), value-based care (median 3.5, IQR 2,6), and health disparities (median 4, IQR 2,5). This survey identified top HM research priorities including systems-based topics and health disparities, aligning with HM’s identity as a specialty focused on improving systems of care and inequities in care.

## INTRODUCTION

Hospital medicine (HM) is a well-established yet still evolving clinical field.^1,2^ Although HM carries a strong identity for value and systems-based care, quality, and equity,^3^ the HM research footprint has lagged.^1,4^ In a survey of 266 US academic hospitalists, less than half reported having a mentor or having presented at a national meeting.^5^ To date, several studies have reported the state of HM research. A 2018 survey found low levels of research activity both in a number of faculty with research funding (12%) and funding dollars (29% of programs received more than $1,000,000 in research funding).^6^ Further, only five research fellowships were identified; lack of funding was the leading barrier to establishing HM research fellowships.^6^ A 2023 follow-up survey found only 2% of hospitalists reported spending 50% or more of their time on research.^7^ The findings in these two studies were supported by a similar national survey of HM programs.^4^ For comparison, similar numbers of hospitalists and cardiologists exist (44,037 vs. 47,225),^8^ but over 10% of cardiologist faculty hold NIH funding.^9^ One barrier to increasing HM research participation is limited funding for HM research.^10^ Further, the breadth of clinical focus within HM makes it difficult to have a specific National Institute of Health home. A multilayered approach is required to address these challenges and more clearly define a HM research agenda. One important component is increasing advocacy for targeted federal funding mechanisms, which requires clearly defined HM research priorities and expertise. A study published in 2020 identified 11 patient-centered HM research priorities that included shared decision-making and improving communication across care settings and care teams.^11^ Given the ongoing lag in HM research training, funding, and professional advancement, and the gap with respect to prioritization among HM clinicians and leaders, additional efforts to identify and disseminate top research topics are critical. Therefore, we aimed to further define this study agenda leveraging a survey of national HM leaders to identify their research priorities.

## METHODS

In 2020, the Society of Hospital Medicine (SHM) Research Committee formed a sub-committee of HM researchers with diverse research expertise and funding support, charged with defining the HM research agenda priorities. The University of Chicago institutional review board deemed this study exempt.

The HM Research subcommittee developed a survey outlining potential key HM research priorities via an iterative, consensus-based approach, using input from both committee members and HM leaders outside of the committee. The final result was a 20-item survey with eight primary domains of research priorities (e.g., patient safety, health disparities), each with four to eight subdomains (e.g., patient safety subdomains: diagnostic error, medication safety). To account for additional domains not explicitly listed, there was an option for “other” for respondents to identify additional primary domains and subdomains for ranking. (Supporting Information S1: Appendix A) For each primary domain and subdomain, the survey asked respondents to rank each item in the respective domain against each other on a scale, from 1 being the highest priority to “x” (total number of items per domain/subdomain). This rank methodology was intentionally chosen instead of a scale-based response for level of importance to avoid a ceiling effect (where all domains are highly ranked), which would prevent meaningful comparison. The survey also included questions on demographics and professional appointments.

We used purposeful sampling to identify key HM leaders. To obtain the most diverse responses we used snowball sampling to enable inclusion of a wide range of hospitalists, not just researchers, recognizing this might affect the response rate. We leveraged an existing distribution list from the State of HM research to identify leaders in HM (e.g., division chiefs).^7^ In addition, we identified other HM leaders involved in SHM (e.g., committee chairs) and the Journal of Hospital Medicine (JHM; i.e., editors). We also identified leaders within organizations external to SHM with high HM membership (e.g., Society of General Internal Medicine (SGIM) and Journal of General Internal Medicine [JGIM]).^12^ Survey respondents could provide contact information for additional HM senior faculty, research fellows, and junior faculty, who were then invited to complete the survey. The survey was sent electronically using REDCap between September 2023 and April 2024 with up to 3 weekly follow-up emails to nonresponders.

Descriptive statistics were used to summarize demographics and professional characteristics and rank primary and subdomains. Median (interquartile range [IQR]) was reported for continuous variables (e.g., domain ranks) and frequency (percentage) for categorical variables (e.g., respondent characteristics). Kruskal–Wallis tests (continuous variables) and *χ*^2^ tests (categorical variables) were performed, with Bonferroni correction for multiple comparisons.

Given the anticipated high nonresponse rate among nonresearcher HM leaders, we evaluated response bias by classifying participants as “respondents” (partial/full survey completion) or “non-respondents” (survey not opened/started). Academic rank and institution type were compared between groups. Contingency tables were reviewed to ensure data completeness and validity.

Analyses were performed using R statistical software (R Foundation for Statistical Computing), with significance established at *p* < .05.

## RESULTS

Of the 239 surveys sent, 81 were at least partially completed (33.9%) and 66 (27.6%) were fully completed. *Overall, 46% of respondents were male and 33% were female*. Most respondents reported White race (43/81, 53%), and working at academic institutions (46/81, 57%) versus nonacademic or hybrid academic settings (43%). Half of respondents (51% 41/81) reported hybrid roles, encompassing various combinations of clinical, research, administrative, and educational responsibilities. Additionally, 15 respondents (19%) identified as associate professors, 22 (27%) were assistant professors, and 14 (17%) were professors. Overall, 18 (44%) had HM leadership roles while 28 (35%) were research fellows or junior faculty (Table 1).

Comparisons between respondents and nonrespondents revealed significant differences in institution type, with academic institutions overrepresented among respondents (56% vs. 44%, *p* = .03). No statistically significant differences were found in academic rank, suggestive of similar distributions of professors, associate professors, and assistant professors between groups.

Across the eight primary research topic domains, the priority rankings ranged from median scores of two to seven. The top three ranked primary research topic domains were “innovation in care delivery” (median 2.0, [1,3]), “value-based care” (median 3.5, [2,6]), and “health disparities” (median 4.0 [2,5]). Within these top three primary domains, top ranked subdomains were: “Informatics” and “transitions of care” (median 3 for each); “high-value care” (median 1); and “social determinants of health” and “trust in patient/clinician relationship” (median 2 for both), respectively. Among the “other” domains, the most common write-in response was research mentorship/early career support. All rankings of primary domains and subdomains and the list of all write-in responses are available in Table 2.

There were some notable differences in primary domain and subdomain ranking by respondent demographics. For example, male respondents ranked the primary domain of “HM Specific Conditions/Diseases” as a greater priority than female respondents (median 2 [1,5] *vs*. 5 [4,7]). Similarly, differences were observed in the ranking of the “Patient Experience” domain by respondents’ institution type: those from academic institutions ranked it a lower priority than nonacademic institutions (median 5 [4,6] *vs*. 3 [2,5]). Additionally, the primary domain of “Patient Safety” differed by years in practice: respondents with fewer years in practice (1–5 years) ranked a lower priority (median 5 [4,6]) than those with over 20 years of experience (median 3 [2,4]). (Supporting Information S2: Appendix S2). However, the number of respondents within some stratified demographic analyses was relatively low, limiting our ability to test the significance of between-group differences.

## CONCLUSIONS

This purposeful survey of key HM research leaders contributes to the identification of top HM research priorities. Systems-based topics (e.g., innovations in care delivery and value-based care) and health disparities were the highest ranked priority areas, which aligns with our identity as a specialty that is focused on improving systems of care and addressing inequities in care.

To advance HM research and funding, we will share these prioritized topics with the HM community for discussion and refinement. We will also advocate for increased and/or aligning existing federal and society funding emphasizing healthcare systems-based rather than organ-specific mechanisms.^13^ For example, given the adjacent alignment of several identified priorities (i.e., addressing inequities in care) with the recently launched NIH Communities Advancing Research Equity for Health (CARE for Health Initiative), these results could be used to advocate for expansion of these funds to encompass HM in addition to primary care based research. Additionally, these results could be shared with NIH-adjacent federal funding sources that have more traditionally supported HM-based research (e.g., the Agency for Healthcare Research and Quality [AHRQ]). The long-term goal is to support a clear research agenda that expands training and mentorship opportunities, such as HM research fellowships.^10^ A stronger research workforce and increased funding will position HM to advance the science underpinning the specialty.

While this report provides critical prioritization of HM research topics, there are some limitations. First, our response rate was expectedly low. However, our comparison of respondents versus nonrespondents demonstrated an expected over-representation by academic institutions, which is reflective of the HM research community. Though we allowed free-text responses, the study was also limited by the available responses being generated by a group of HM researchers. Furthermore, the survey respondents largely included HM leaders. Our findings are reflective of a more circumscribed highly vested perspective, rather than broader, multi-informant perspective. This study focused on adult, but not pediatric, HM research priorities. Although some existing research has specifically targeted aspects of pediatric HM research,^14,15^ prioritization of pediatric HM research topics requires additional exploration. Further, due to resource constraints, we employed a consensus-based approach with expert input from a subset of HM research leaders (i.e., those within the SHM Research Committee) to develop the survey used in this study, and utilized a purposeful sample of survey respondents similar to other studies.^6,7^ Additional work to follow-up our study could use other methodology, such as the Delphi method to increase specificity and help guide advocacy efforts for federal funding. Lastly, while the aim of this study was to highlight key prioritized HM research topics, this does not diminish the importance of HM research topics not included within the identified top priorities.

In summary, these findings represent an important first step in developing a research agenda for HM. Future efforts could build on this report and other similar work, such as the HM geriatric research agenda by Wald et al.,^16^ to identify broader HM research priorities. Having clear priorities will help to establish the areas in which hospitalists are the subject matter experts and leaders that should create the evidence for how we care for our patients today and in the future. Other future efforts include disseminating these findings to the HM community at large, including society leadership (SHM, SGIM), journals (JHM, JGIM), and funders (National Institutes of Health, Agency for Healthcare Quality and Research, Patient Centered Outcomes Research Institute). These data can be leveraged to advocate for funding to support identified HM research priorities.

## Supplementary Material

Appendix Figure A: Society of Hospital Medicine Research Committee Research Priority Survey Top Hospital Medicine Research Topics

Appendix B: Appendix Table: Topic Preferences Stratified by Academic Rank, Institution Type, Professional Role

Strobe Checklist

## Figures and Tables

**TABLE 1:** Respondent characteristics (*N* = 81).

Category	N(%)
Gender	
Female	27 (33.3)
Male	37 (45.7)
Prefer not to state/not available	17 (21.0)
Race	
Asian	13 (16.0)
Black or African American	3 (3.7)
White or Caucasian	43 (53.1)
Other	22 (27.2)
Ethnicity	
Hispanic, Latino, Latina, Latinx	2 (2.5)
Not Hispanic, Latino, Latina, Latinx	56 (69.1)
Prefer not to respond/Not answered	23 (28.4)
Institution type	
Academic	46 (56.8)
Hybrid Academic + Community	4 (4.9)
Hybrid Academic + Community + Veterans Affairs	1 (1.2)
Hybrid Academic + Veterans Affairs	9 (11.1)
Veterans Affairs	5 (6.2)
Other	2 (2.5)
Not answered	14 (17.3)
Professional role	
Hybrid (administrative; education; clinical; +/or research)	41 (50.6)
Primarily administrative	6 (7.4)
Primarily clinical	3 (3.7)
Primarily research	17 (21.0)
Not answered	14 (17.3)
Leadership role	
Department Chair	2 (2.5)
Department Quality Officer	1 (1.2)
Division/Section Chief	15 (18.5)
Research Fellowship Director	3 (3.7)
Other	25 (30.9)
Not answered	35 (43.2)
Academic Rank	
Adjunct Professor	4 (4.9)
Instructor	1 (1.2)
Assistant professor	22 (27.2)
Associate professor	15 (18.5)
Professor	14 (17.3)
Emeritus	1 (1.2)
Other	10 (12.3)
Not answered	14 (17.3)

**TABLE 2:** Priority ranking of domains and subdomains (1 [highest]).^a^

Domain	Subdomain	Median	IQR
1. Innovation in care delivery		2.00	1.00–3.00
1.	Informatics^b^	3.00	2.00–5.00
2.	Transition of care^b^	3.00	2.00–5.00
3.	Care coordination	4.00	2.25–6.75
4.	Telemedicine	4.50	3.75–6.25
5.	Hospital at home	5.00	2.00–6.00
6.	Multi-disciplinary collaboration	5.00	3.00–7.00
7.	Readmission reduction	5.00	3.00–8.00
8.	Other innovation topic^b^	7.00	6.00–8.00
9.	Advanced practice provider integration^b^	7.00	6.00–8.00
10.	Comanagement	8.00	6.00–9.00
2. Value-based care		3.50	2.00–6.00
1.	High value care	1.00	1.00–2.00
2.	Value-based payment	3.00	2.00–3.25
3.	Choosing wisely^®b^	3.00	2.00–4.00
4.	Population health^b^	3.00	2.00–4.00
5.	Other value-based care topic	5.00	5.00–5.00
3. Health disparities		4.00	2.00–5.00
1.	Social determinants of health	2.00	1.00–2.00
2.	Trust in patient/clinician relationship	2.00	2.00–3.00
3.	New payment systems^a^	3.00/	2.00–4.00
4.	Personalized medicine^a^	3.00	2.00–4.00
5.	Other disparity topic	5.00	4.00–5.00
4. Hospital medicine-specific conditions/diseases		4.00	2.00–6.00
1.	Other hospital medicine specific condition/disease^b^ (e.g., CHF, COPD, Stroke, etc)^c^	2.00	1.00–3.00
2.	Top diagnoses for 30-day readmission^b^	2.00	1.00–3.00
3.	Sepsis	2.00	2.00–3.00
4.	Venous thromboembolism	3.00	2.75–4.00
5.	COVID-19	4.00	4.00–5.00
5. Patient safety		4.00	3.00–5.75
1.	Diagnostic error	2.00	1.00–3.00
2.	Medication safety	3.00	2.00–5.00
3.	Healthcare-associated infections	4.00	2.00–6.00
4.	Delirium prevention^b^	4.00	3.00–00
5.	Antibiotic stewardship^b^	4.00	3.00–6.00
	Appropriate device use	5.50	4.00–8.00
6.	Appropriate opioid use	6.00	4.00–6.00
8.	Fall prevention	7.00	5.00–8.00
9.	Other patient safety topic	9.00	7.50–9.00
6. Patient experience		5.00	4.00–6.00
1.	Communication	1.00	1.00–2.00
2.	Health literacy	2.00	1.00–3.00
3.	Technology literacy^b^	3.00	2.00–4.00
4.	Patient education^b^	3.00	2.00–4.00
5.	Other patient experience topic	5.00	4.50–5.00
7. Methodologies used in hospital medicine research		6.00	4.00–7.00
1.	Implementation science	2.00	1.00–3.00
2.	AI/Machine learning	2.00	1.00–4.00
3.	Pragmatic trials	3.00	2.00–4.00
4.	Comparative effectiveness research	4.00	3.00–5.00
5.	Qualitative research	5.00	4.00–.00
6.	Translational research	6.00	4.00–7.00
7.	Survey research	6.00	5.00–7.00
8.	Other methodologies	8.00	6.25–8.00
8. Other^c^		7.00	3.75–8.00

Abbreviations: CHF, congestive heart failure; COPD, chronic obstructive pulmonary disease.

a The table reports data first by the ranking of the domains highest priority and then the highest ranking within each subdomain (median rank; rank of 1 is highest).

b Equal rank.

c Majority of other hospital medicine conditions was CHF. Domain priorities identified by the “Other” option: environmental health, geriatric hospital medicine, transition from pediatric to adult care, care of patients with high needs, behavioral health, provider safety, provider well-being, quality measurement, provider assessment, research mentorship/early career support (e.g., protected time), clinical decision making, and AI.
