# Supplementary material for: Research priorities for adult hospital medicine: A survey of US hospital medicine leaders
Source: J Hosp Med. 2025 Apr 20;20(11):1206–11. doi: 10.1002/jhm.70053 (PMC12353494; doi:10.1002/jhm.70053)
Supplement: Supplementary file 1 — Supporting information. [file JHM-20-1206-s003.docx]

**Appendix Figure A:** Society of Hospital Medicine Research Committee Research Priority Survey

Top Hospital Medicine Research Topics

**Please rank order the 8 hospital medicine research topics listed below by placing each one in order from highest priority [1] to lowest priority [8]).** When ranking think about the state of hospital medicine research as a whole and which of these broad topics should be of highest priorities for funding, attention, policy, etc. After completing this section, where the focus is on the 'big picture' topics, you will then be asked to rank several sub-topics within each of "big picture' topics.

|  | 1  (highest) | | 2 | 3 | 4 | 5 | 6 | 7 | 8  (lowest) |
| --- | --- | --- | --- | --- | --- | --- | --- | --- | --- |
| Health Disparities |  |  |  |  |  |  |  |  |  |
| Hospital Medicine-specific Conditions/Diseases |  |  |  |  |  |  |  |  |  |
| Innovation in Care Delivery |  |  |  |  |  |  |  |  |  |
| Methodologies used in Hospital Medicine research |  |  |  |  |  |  |  |  |  |
| Patient Experience |  |  |  |  |  |  |  |  |  |
| Patient Safety |  |  |  |  |  |  |  |  |  |
| Value-Based Care |  |  |  |  |  |  |  |  |  |
| Other Hospital Medicine Research Topic |  |  |  |  |  |  |  |  |  |

**Please rank the following sub-topics within Health Disparities from 1 (highest priority) to 5 (lowest priority)**

|  | 1  (highest) | 2 | 3 | 4 | 5  (lowest) |
| --- | --- | --- | --- | --- | --- |
| Personalized medicine |  |  |  |  |  |
| Trust in patient/clinician relationship |  |  |  |  |  |
| Social determinants of health |  |  |  |  |  |
| New payment systems |  |  |  |  |  |
| Other health disparities topic |  |  |  |  |  |

**Please rank the following sub-topics within Hospital Medicine Specific Conditions/ Diseases from 1 (highest priority) to 5 (lowest priority)**

|  | 1  (highest) | 2 | 3 | 4 | 5  (lowest) |
| --- | --- | --- | --- | --- | --- |
| Top diagnoses for 30-day readmission |  |  |  |  |  |
| Disease specific: Sepsis |  |  |  |  |  |
| Disease specific: COVID-19 |  |  |  |  |  |
| Disease specific: Venous thromboembolism (VTE) |  |  |  |  |  |
| Other hospital medicine specific condition/disease  (e.g., CHF, COPD, Strokes, etc) |  |  |  |  |  |

**Please rank the following sub-topics within Innovation in Care Delivery from 1 (highest priority) to 10 (lowest priority)**

|  | 1  (highest) | 2 | 3 | 4 | 5 | 6 | 7 | 8 | 9 | 10  (lowest) |
| --- | --- | --- | --- | --- | --- | --- | --- | --- | --- | --- |
| Transitions of care |  |  |  |  |  |  |  |  |  |  |
| Readmission reduction |  |  |  |  |  |  |  |  |  |  |
| Hospital at home |  |  |  |  |  |  |  |  |  |  |
| Tele-hospitalists/telemedicine |  |  |  |  |  |  |  |  |  |  |
| Informatics- electronic health record/health Information technology |  |  |  |  |  |  |  |  |  |  |
| Care coordination |  |  |  |  |  |  |  |  |  |  |
| Multi-disciplinary collaboration |  |  |  |  |  |  |  |  |  |  |
| Advanced practice provider integrations |  |  |  |  |  |  |  |  |  |  |
| Co-management |  |  |  |  |  |  |  |  |  |  |
| Other innovation in care delivery topic |  |  |  |  |  |  |  |  |  |  |

**Please rank the following sub-topics within Hospital Medicine Research Methodologies from 1 (highest priority) to 8 (lowest priority)**

|  | 1  (highest) | 2 | 3 | 4 | 5 | 6 | 7 | 8  (lowest) |
| --- | --- | --- | --- | --- | --- | --- | --- | --- |
| Survey research |  |  |  |  |  |  |  |  |
| Implementation science |  |  |  |  |  |  |  |  |
| Artificial intelligence/machine learning |  |  |  |  |  |  |  |  |
| Translational research |  |  |  |  |  |  |  |  |
| Qualitative research |  |  |  |  |  |  |  |  |
| Comparative effectiveness research |  |  |  |  |  |  |  |  |
| Pragmatic trials |  |  |  |  |  |  |  |  |
| Other hospital medicine research methodology |  |  |  |  |  |  |  |  |

**Please rank the following sub-topics within Patient Experience from 1 (highest priority) to 5 (lowest priority)**

|  | 1  (highest) | 2 | 3 | 4 | 5  (lowest) |
| --- | --- | --- | --- | --- | --- |
| Communication |  |  |  |  |  |
| Health literacy |  |  |  |  |  |
| Technology literacy |  |  |  |  |  |
| Patient education |  |  |  |  |  |
| Other patient experience topic |  |  |  |  |  |

**Please rank the following sub-topics within Patient Safety from 1 (highest priority) to 9 (lowest priority)**

|  | 1  (highest) | 2 | 3 | 4 | 5 | 6 | 7 | 8 | 9 (lowest) |
| --- | --- | --- | --- | --- | --- | --- | --- | --- | --- |
| Antibiotic stewardship |  |  |  |  |  |  |  |  |  |
| Healthcare associated infections |  |  |  |  |  |  |  |  |  |
| Appropriate device use |  |  |  |  |  |  |  |  |  |
| Fall prevention |  |  |  |  |  |  |  |  |  |
| Delirium prevention |  |  |  |  |  |  |  |  |  |
| Appropriate opioid use |  |  |  |  |  |  |  |  |  |
| Medication safety |  |  |  |  |  |  |  |  |  |
| Diagnostic error |  |  |  |  |  |  |  |  |  |
| Other patient safety topic |  |  |  |  |  |  |  |  |  |

**Please rank the following sub-topics within Value Based Care from 1 (highest priority) to 5 (lowest priority)**

|  | 1  (highest) | 2 | 3 | 4 | 5  (lowest) |
| --- | --- | --- | --- | --- | --- |
| High-value care |  |  |  |  |  |
| Choosing wisely |  |  |  |  |  |
| Population health |  |  |  |  |  |
| Value-based care (payment model) |  |  |  |  |  |
| Other value based care topic |  |  |  |  |  |

**Anything else to add?** Any other comments on priorities for research in hospital medicine:

**Recommendations of individuals to whom we should send the survey**

Please provide name(s) and email address(es) of thought leaders to whom you suggest we should send the survey:

Please provide name(s) and email address(es) of fellows and/or junior faculty to whom you suggest we send the survey:

**Demographics:** Please complete the following to allow us to understand the diversity of participants completing the survey and diversity of sites being represented

If US: State/District/Territory you work in. If non-US- Country: ___________________________________

| Academic Rank | Hospital Medicine Research Fellow |
| --- | --- |
|  | Instructor |
|  | Assistant Professor |
|  | Associate Professor |
|  | Adjunct Professor |
|  | Professor |
|  | Emeritus |
|  | Other |

| Years in practice | Less than 1 year |
| --- | --- |
|  | 1-5 years |
|  | 6-10 years |
|  | 11-15 years |
|  | 16-20 years |
|  | Greater than 20 years |

| Institution type | Academic |
| --- | --- |
|  | Community |
|  | VA |
|  | Hybrid Academic + Community |
|  | Hybrid Academic + VA |
|  | Hybrid Community + VA |
|  | Hybrid Academic + Community + VA |
|  | Other |

| Professional role | Primarily clinical |
| --- | --- |
|  | Primarily research |
|  | Primarily administrative |
|  | Primarily education |
|  | Hybrid clinical + research |
|  | Hybrid clinical + administrative |
|  | Hybrid clinical + education |
|  | Hybrid research+ administrative |
|  | Hybrid research + education |
|  | Hybrid clinical + research + administrative |
|  | Hybrid research + administrative + education |
|  | Hybrid administrative + education + clinical |
|  | Hybrid clinical + research + administrative + education |
|  | Other |

| Leadership Roles- please check the primary role you hold if any | Department Chair |
| --- | --- |
|  | Division/Section Chief |
|  | Chief Quality Officer |
|  | Department Quality Officer |
|  | Division/Section Chief Quality Officer |
|  | Research Fellowship Director |
|  | Other |

| Gender | Male |
| --- | --- |
|  | Female |
|  | Transgender Male |
|  | Transgender Female |
|  | Non-binary/non-conforming |
|  | Other |

| Race | Black or African American |
| --- | --- |
|  | White |
|  | Asian |
|  | American Indian or Alaska Native |
|  | Native Hawaiian or Other Pacific Islander |
|  | Multiple |
|  | Other |

| Ethnicity | Hispanic, Latino, Latina, Latinx |
| --- | --- |
|  | Not Hispanic, Latino, Latina, Latinx |
|  | Other |
|  | Prefer not to respond |
