# Supplementary material for: Research priorities for adult hospital medicine: A survey of US hospital medicine leaders
Source: J Hosp Med. 2025 Apr 20;20(11):1206–11. doi: 10.1002/jhm.70053 (PMC12353494; doi:10.1002/jhm.70053)
Supplement: Supplementary file 2 — Supporting information. [file JHM-20-1206-s002.docx]

**Appendix B:** Appendix Table: Topic Preferences Stratified by Academic Rank, Institution Type, Professional Role

| **Appendix Table: Topic Preferences Stratified by Academic Rank, Institution Type, Professional Role** | | | | | |  |
| --- | --- | --- | --- | --- | --- | --- |
| **Median (Min, Max) and Kruskal-Wallis Test Results** | | | | | |  |
| **Topic** | **Category** | **Subgroup (N)** | **Median (IQR (p25, p75))** |  |  |  |
| Health Disparities | Academic Rank | Early-Career (34) | 3.5 (2.25, 5.25) |  |  |  |
|  |  | Mid-Senior (33) | 5.0 (3.0, 7.0) |  |  |  |
|  |  | Not Answered (14) | 5.0 (3.5, 6.5) |  |  |  |
|  | Institution Type | Academic (60) | 4.0 (2.5, 5.5) |  |  |  |
|  |  | Non-Academic (7) | 5.0 (4.0, 7.0) |  |  |  |
|  |  | Not Answered (14) | 5.0 (3.5, 6.5) |  |  |  |
|  | Professional Role | Administrative (6) | 5.0 (3.0, 6.0) |  |  |  |
|  |  | Clinical (3) | 3.0 (2.0, 3.5) |  |  |  |
|  |  | Hybrid (41) | 4.0 (2.5, 6.5) |  |  |  |
|  |  | Research (17) | 4.0 (2.5, 5.5) |  |  |  |
|  |  | Not Answered (14) | 5.0 (3.5, 6.5) |  | | |
|  | | | | | |  |
| Hospital Medicine Specific Conditions/ Diseases | Academic Rank | Early-Career (34) | 4.0 (2.5, 6.0) |  |  |  |
|  |  | Mid-Senior (33) | 4.0 (2.5, 6.0) |  |  |  |
|  |  | Not Answered (14) | 4.5 (2.75, 5.75) |  |  |  |
|  | Institution Type | Academic (60) | 4.0 (2.5, 6.0) |  |  |  |
|  |  | Non-Academic (7) | 4.0 (2.5, 5.0) |  |  |  |
|  |  | Not Answered (14) | 4.5 (2.75, 5.75) |  |  |  |
|  | Professional Role | Administrative (6) | 2.0 (2.0, 4.0) |  |  |  |
|  |  | Clinical (3) | 2.0 (1.5, 5.0) |  |  |  |
|  |  | Hybrid (41) | 4.0 (2.5, 6.0) |  |  |  |
|  |  | Research (17) | 4.0 (2.5, 6.0) |  |  |  |
|  |  | Not Answered (14) | 4.5 (2.75, 5.75) |  | | |
|  | | | | | |  |
| Innovation in Care Delivery | Academic Rank | Early-Career (34) | 2.0 (1.5, 4.5) |  |  |  |
|  |  | Mid-Senior (33) | 2.0 (1.5, 4.5) |  |  |  |
|  |  | Not Answered (14) | 3.0 (2.0, 4.5) |  |  |  |
|  | Institution Type | Academic (60) | 2.0 (1.5, 4.5) |  |  |  |
|  |  | Non-Academic (7) | 2.5 (1.75, 4.75) |  |  |  |
|  |  | Not Answered (14) | 3.0 (2.0, 4.5) |  |  |  |
|  | Professional Role | Administrative (6) | 1.0 (1.0, 2.0) |  |  |  |
|  |  | Clinical (3) | 3.0 (2.0, 3.0) |  |  |  |
|  |  | Hybrid (41) | 2.0 (1.5, 4.5) |  |  |  |
|  |  | Research (17) | 2.0 (1.5, 4.0) |  |  |  |
|  |  | Not Answered (14) | 3.0 (2.0, 4.5) |  | | |
|  | | | | | |  |
| Methodologies used in Hospital Medicine Research | Academic Rank | Early-Career (34) | 5.5 (3.25, 6.75) |  |  |  |
|  |  | Mid-Senior (33) | 7.0 (4.0, 8.0) |  |  |  |
|  |  | Not Answered (14) | 4.5 (2.75, 5.75) |  |  |  |
|  | Institution Type | Academic (60) | 6.0 (3.5, 7.5) |  |  |  |
|  |  | Non-Academic (7) | 5.5 (3.25, 6.25) |  |  |  |
|  |  | Not Answered (14) | 4.5 (2.75, 5.75) |  |  |  |
|  | Professional Role | Administrative (6) | 6.5 (6.25, 7.75) |  |  |  |
|  |  | Clinical (3) | 5.0 (3.5, 6.0) |  |  |  |
|  |  | Hybrid (41) | 6.0 (3.5, 7.5) |  |  |  |
|  |  | Research (17) | 6.0 (3.5, 7.0) |  |  |  |
|  |  | NA (14) | 4.5 (2.75, 5.75) |  | | |
|  | | | | | |  |
| Patient Experience | Academic Rank | Early-Career (34) | 5 (3.0, 6.5) |  |  |  |
|  |  | Mid-Senior (33) | 5 (3.0, 6.5) |  |  |  |
|  |  | Not Answered (14) | 3 (2.0, 5.5) |  |  |  |
|  | Institution Type | Academic (60) | 6 (3.5, 7.0) |  |  |  |
|  |  | Non-Academic (7) | 2 (1.5, 3.5) |  |  |  |
|  |  | Not Answered (14) | 3 (2.0, 5.5) |  |  |  |
|  | Professional Role | Administrative (6) | 4 (3.5, 5.0) |  |  |  |
|  |  | Clinical (3) | 4 (4.0, 5.5) |  |  |  |
|  |  | Hybrid (41) | 5 (3.0, 6.5) |  |  |  |
|  |  | Research (17) | 6 (3.5, 7.0) |  |  |  |
|  |  | Not Answered (14) | 3 (2.0, 5.5) |  | | |
|  | | | | | |  |
| Patient Safety | Academic Rank | Early-Career (34) | 4.0 (3.0, 5.5) |  |  |  |
|  |  | Mid-Senior (33) | 4.0 (2.5, 5.5) |  |  |  |
|  |  | Not Answered (14) | 5.5 (3.75, 6.25) |  |  |  |
|  | Institution Type | Academic (60) | 4.0 (2.5, 5.5) |  |  |  |
|  |  | Non-Academic (7) | 3.5 (2.75, 5.25) |  |  |  |
|  |  | Not Answered (14) | 5.5 (3.75, 6.25) |  |  |  |
|  | Professional Role | Administrative (6) | 3.5 (3.25, 4.25) |  |  |  |
|  |  | Clinical (3) | 5.0 (5.0, 6.0) |  |  |  |
|  |  | Hybrid (41) | 4.0 (2.5, 5.5) |  |  |  |
|  |  | Research (17) | 5.0 (3.5, 5.5) |  |  |  |
|  |  | Not Answered (14) | 5.5 (3.75, 6.25) |  | | |
|  | | | | | |  |
| Value Based Care | Academic Rank | Early-Career (34) | 4.0 (2.5, 6.0) |  |  |  |
|  |  | Mid-Senior (33) | 3.0 (2.0, 6.0) |  |  |  |
|  |  | Not Answered (14) | 4.0 (2.5, 5.5) |  |  |  |
|  | Institution Type | Academic (60) | 4.0 (2.5, 6.0) |  |  |  |
|  |  | Non-Academic (7) | 5.0 (3.0, 7.0) |  |  |  |
|  |  | Not Answered (14) | 4.0 (2.5, 5.5) |  |  |  |
|  | Professional Role | Administrative (6) | 3.5 (2.25, 5.25) |  |  |  |
|  |  | Clinical (3) | 6.0 (4.0, 6.0) |  |  |  |
|  |  | Hybrid (41) | 4.0 (2.5, 6.5) |  |  |  |
|  |  | Research (17) | 4.0 (2.5, 5.5) |  |  |  |
|  |  | Not Answered (14) | 4.0 (2.5, 5.5) |  | | |
|  | | | | | |  |
| Other Priorities | Academic Rank | Early-Career (34) | 8.0 (5.5, 8.5) |  |  |  |
|  |  | Mid-Senior (33) | 8.0 (4.5, 8.0) |  |  |  |
|  |  | Not Answered (14) | 7.0 (4.0, 7.5) |  |  |  |
|  | Institution Type | Academic (60) | 8.0 (4.5, 8.5) |  |  |  |
|  |  | Non-Academic (7) | 8.0 (8.0, 8.0) |  |  |  |
|  |  | Not Answered (14) | 7.0 (4.0, 7.5) |  |  |  |
|  | Professional Role | Administrative (6) | 7.5 (7.25, 7.75) |  |  |  |
|  |  | Clinical (3) | 7.0 (6.5, 7.5) |  |  |  |
|  |  | Hybrid (41) | 8.0 (4.5, 8.5) |  |  |  |
|  |  | Research (17) | 6.0 (4.5, 7.5) |  |  |  |
|  |  | NA (17) | 7.0 (4.0, 7.5) |  |  |  |
| **Academic institutions** **defined as**: *Academic, Hybrid Academic + Community, Hybrid Academic + VA, and Hybrid Academic + Community + VA.* **Non-Academic institutions** **defined as:** *Community, VA, Hybrid Community + VA, and Other.* | | | |  |  |  |
